# A case of advanced atrioventricular block after gynecological surgery

**DOI:** 10.1186/s40981-020-00387-8

**Published:** 2020-10-09

**Authors:** Takuzou Kitazawa, Mitsuru Ida, Masahiko Kawaguchi

**Affiliations:** Department of Anesthesiology, Nara Medical University, 840 Shijo-cho, Kashihara, Nara, 634-8522 Japan

To the Editor,

Advanced atrioventricular block (AVB) after non-cardiac surgery is rare, but occasionally leads to serious complications such as syncope. After the onset of AVB, the causes are investigated and treatment is selected according to the symptoms [1]. We report a case of advanced AVB in the patient without any comorbidities after gynecological surgery that may have been triggered by postoperative nausea.

Open uterine myomectomy was planned for a 39-year-old healthy woman. Her electrocardiographic findings were normal range and there was no history of loss of consciousness before surgery. After the uneventful anesthesia induction, her trachea was intubated. Then, a mixture of 0.375% ropivacaine (total dose 40 mL) was injected bilaterally into the fascial plane with ultrasound. Intraoperatively, general anesthesia was maintained with propofol, fentanyl (250 mcg), remifentanil, and rocuronium and no anti-emetics were administrated. After the uneventful procedure, the trachea was extubated following administration of sugammadex, and the patient was transferred to the general ward with an Aldrete score of 10 [2]. On postoperative day 1, 17 h and 14 min after surgery, she first complained of nausea and suddenly lost consciousness following oculogyric crisis and rigidity of her upper extremities. She then spontaneously regained consciousness in approximately 25 s (Fig. 1). The same loss of consciousness following nausea occurred three times within 2 h after she first lost consciousness. A consulted cardiologist made a diagnosis of advanced AVB. Her blood sample, echocardiography, and electrocardiogram ruled out electrolyte abnormalities and myocardial ischemia. Then, with the administration of antiemetic drug, a temporary transvenous cardiac pacing leads were placed. After that, the patient followed an uneventful course without nausea and developing advanced AVB and was discharged from our hospital with removal of her temporary pacing lead on postoperative day 10.

**Fig. 1:**
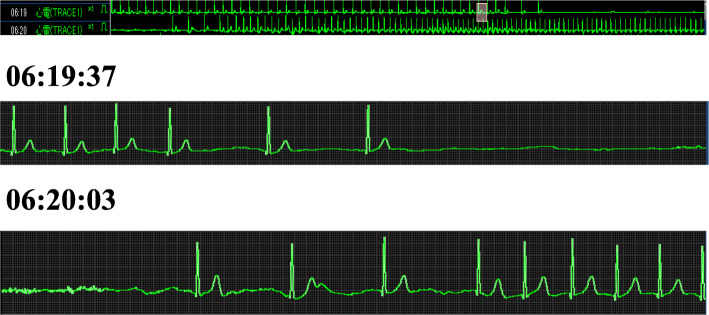
Electrocardiogram recorded in the ward 17 hours and 14 minutes after surgery. Trend recording during the first episode of bradycardia (top), at the onset of bradycardia and cardiac arrest (middle) and spontaneous recovery approximately in 23 s (bottom)

It has been reported that nausea and vomiting can stimulate the vagal nerve to induce bradycardia and AVB [3]. Recently, however, the vagal score was developed to determine the involvement of the vagal nerve in AVB using electrocardiogram waveforms with a total score ≥ 3 strongly suggesting a vagal mediated mechanism [4]. In our case, following four items were positive with the total score of 4; no AVB or intraventricular conduction disturbance on the baseline electrocardiogram, PR prolongation immediately before paroxysmal AVB, sinus slowing immediately before paroxysmal AVB, resumption of atrioventricular conduction with PP shortening. Additionally, the fact that multiple advanced AVBs following nausea perhaps indicate that the vagal nerve was stimulated by nausea [3]. Furthermore, temporary pacing lead was placed because of her multiple loss of consciousness. In order to prevent postoperative nausea and vomiting, general anesthesia was maintained with propofol; however, prophylactic administration of anti-emetics might reduce the incidence and the severity of postoperative nausea [5]. Postoperative advanced AVB occurs in non-cardiac surgery; however, early detection of the cause can help explain the mechanism of advanced AVB to patient. Furthermore, because postoperative nausea and vomiting may have caused advanced AVB by stimulating the vagal nerve, more careful management strategy to prevent postoperative nausea and vomiting may have been necessary in patients at high risk for postoperative nausea and vomiting.

## Data Availability

Not applicable
